# Abiotic and Biotic Determinants of Plant Diversity in Aquatic Communities Invaded by Water Hyacinth [*Eichhornia* *crassipes* (Mart.) Solms]

**DOI:** 10.3389/fpls.2020.01306

**Published:** 2020-08-25

**Authors:** Hao Wu, Jianqing Ding

**Affiliations:** ^1^ College of Life Sciences, Xinyang Normal University, Xinyang, China; ^2^ School of Life Sciences, Henan University, Kaifeng, China

**Keywords:** biological invasions, diversity index, environmental heterogeneity, stoichiometry, water hyacinth

## Abstract

Rapid global environmental changes could exacerbate the impacts of invasive plants on indigenous plant diversity, especially for freshwater ecosystems characterized by relatively simple plant community structures with low bioresistance. However, the abiotic and biotic determinants of plant diversity in aquatic invaded habitats remain unclear. In this study, we measured four *α*-species diversity indices (the Patrick richness index, Shannon–Wiener diversity index, Simpson diversity index, and Pielou evenness index) in aquatic plant communities invaded by *Eichhornia crassipes* in southern China. We also recorded eight environmental parameters of these communities (longitude, latitude, elevation, dissolved oxygen, water conductivity, nitrate nitrogen, temperature, and precipitation), together with nine biotic traits of *E. crassipes* [abundance, invasion cover, height, total carbon (C) content of the leaves and stems, total nitrogen (N) content of the leaves and stems, and the C:N ratio of leaves and stems]. We then used regression analysis and redundancy analysis (RDA) to determine the dominant factors related to plant diversity. We found that the environment significantly affected *E. crassipes* abundance, height, coverage, stem carbon, and tissue nitrogen, while the leaf C:N stoichiometric ratio was relatively stable. Increasing longitude significantly increased plant diversity, while elevated dissolved oxygen and precipitation slightly improved plant diversity, but increased elevation caused negative effects. *E. crassipes* invasion significantly decreased all four diversity indices. Increases in *E. crassipes* coverage and leaf C:N strongly decreased plant diversity, and increased abundance slightly decreased diversity. Our study indicates that both the changing water environment and the properties of the aquatic invasive plants could have significant impacts on plant diversity. Thus, more attention should be paid to aquatic invasion assessment in lower longitudinal regions with lower native hydrophyte diversity.

## Introduction

Global change, such as climatic warming, nitrogen deposition, and extreme hydrological events, could optimize the ecological and physiological traits of alien species, greatly facilitating their introduction and establishment in new habitats (Wu and Ding, 2019; Probert et al., 2020). As an emerging driver of global change, plant invasions alter species interactions and threaten plant diversity and ecosystem functioning worldwide, causing serious damage to the associated microhabitats (Ding et al., 2008; Sardans et al., 2017; Bernard-Verdier and Hulme, 2019). According to previous studies, many factors that affect the impacts of invasive plants on native plant diversity can be classified into two main categories. One of them is the spatial environmental heterogeneity, which emphasizes abiotic factors such as latitude, nitrogen, climate warming, and precipitation (Pyšek et al., 2005; Wu et al., 2016; Wu et al., 2017; Budzyńska et al., 2019). For instance, warming could increase the interspecific competitiveness of invasive plants, while the increasing latitude might exacerbate their enemy releases at high latitudinal regions with colder climate (Lu et al., 2013; Allen et al., 2017; Wu et al., 2017). The other is invasive plant traits that determine their invasiveness, including phenotypic plasticity, biomass allocation, and fecundity, as invasive plants in invaded habitats usually have the higher abundance, individual size, seed output, and germination than in their origins (Godoy et al., 2012; Rejmánková et al., 2018). Moreover, higher plant diversity could confer biotic resistance against plant invasions at regional scales, while global environmental changes may significantly alter the physiological and/or ecological traits of invasive plants and exacerbate their negative impacts on native cooccurring plants (Byers and Noonburg, 2003; Wu and Ding, 2019). Thus, exploring the abiotic and biotic determinants of plant diversity in invaded ecosystems is critical for predicting plant invasions and conserving native biodiversity under rapid global change (Wu et al., 2017; Rejmánková et al., 2018).

Relative to terrestrial ecosystems, the fluid hydrological connectivity in aquatic ecosystems may accelerate nutrient cycling and transport, which would promote the growth of aquatic invasive plants by providing more suitable environments in which they may spread, particularly for free-floating species such as *Eichhornia crassipes* (Gao et al., 2013; Chappuis et al., 2014; Anufriieva and Shadrin, 2018; Wu and Ding, 2019). Because the species richness of aquatic floras is much lower than that of their terrestrial congeners, aquatic communities may be more vulnerable to alien plant invasions (Santamaría, 2002; Wu et al., 2017). For example, invasion by *E. crassipes* could significantly reduce native plant diversity in freshwater habitats by resource competitions, changing indigenous community structures and occupying vacant niches (Villamagna and Murphy, 2010; Lolis et al., 2020). Furthermore, many aquatic invasive plants respond more dramatically to changes in the water environment than their native cooccurring congeners because as opportunistic species, they usually exhibit higher growth, reproduction, and competitiveness in destabilized aquatic ecosystems with resource fluctuations (*e.g.*, elevated temperature, precipitation, and nitrogen levels) (Collinge et al., 2011; Sorte et al., 2013; You et al., 2014). For instance, a high input of nitrate nitrogen (NO_3_^−^) to Cherry Lake in Australia significantly increased the total biomass accumulation and competitive ability of invasive *Phragmites australis* over those of the accompanying native plants, causing it to largely displace the native species, leading to plant diversity loss in the aquatic ecosystems (Uddin and Robinson, 2018). Finally, both water quality parameters, such as dissolved oxygen and conductivity (Rejmánková et al., 2018; Pulzatto et al., 2019), and superior growth and/or physiological traits (*e.g.*, greater height, abundance, species cover, specific leaf area, nitrogen content, and ratio of carbon/nitrogen) (Fan et al., 2013; Lishawa et al., 2017; Wu et al., 2017) also affect the performance of aquatic invasive plants and their ecological effects. However, studies evaluating the impacts of invasive plants on native plant diversity on the basis of habitat conditions combined with invader properties are relatively rare (but see Lembrechts et al., 2018).

Water hyacinth, *E. crassipes*, which is a free-floating aquatic macrophyte native to South America, has widely invaded freshwater systems in over 50 countries (Villamagna and Murphy, 2010). It has been listed as one of the most detrimental aquatic invasive plant species in southern China (Ding et al., 2006; Ding et al., 2008; Bai et al., 2013). *E. crassipes* often covers the water surface, obstructs rivers, decreases dissolved oxygen, reduces native aquatic plant diversity and even threatens human health by providing a refuge for mosquitoes (Chandra et al., 2006; Villamagna and Murphy, 2010; Zhou et al., 2017). Many recent studies on *E. crassipes* have mainly focused on topics such as its biological control and clonal integration, water purification and the interactions of *E. crassipes* with herbivores (Bownes et al., 2013; Pi et al., 2017; Yu et al., 2019); however, how abiotic factors and the biotic determinants of plant diversity affect the impact of *E. crassipes* is largely unknown. Given that *E. crassipes* shows superior abilities in terms of rapid growth, nutrient absorption, and environmental adaptation (You et al., 2014) and climate change is ongoing (*e.g.*, warming and frequent floods), this invasive plant may expand its northern boundaries to higher latitudes and thus cause larger negative impacts on aquatic plant diversity in these regions (Hoveka et al., 2016; Liu et al., 2017; Wu and Ding, 2019). Therefore, understanding the biotic traits and ecological effects of *E. crassipes* under global environmental changes is critical for accurately predicting its future invasion trends and conserving native biodiversity in aquatic ecosystems.

In this study, we conducted field investigations on aquatic plant communities that were invaded by *E. crassipes* in southern China. We also measured the total carbon and nitrogen contents of *E. crassipes*, and recorded environmental factors in every sampling plot, to examine the determinants of plant diversity in the *E. crassipes-*invaded community.

We hypothesize that heterogeneous environments may significantly affect the impacts of *E. crassipes* on native plant diversity. Specifically, we addressed the following two questions: (1) what are the coupling relationships between the growth and nutrient contents of *E. crassipes* and the environment, and (2) which abiotic (environmental) and biotic (species-specific) factors determine aquatic plant diversity in invaded communities?

## Materials and Methods

### Field Survey and Data Collection

**Figure 1:**
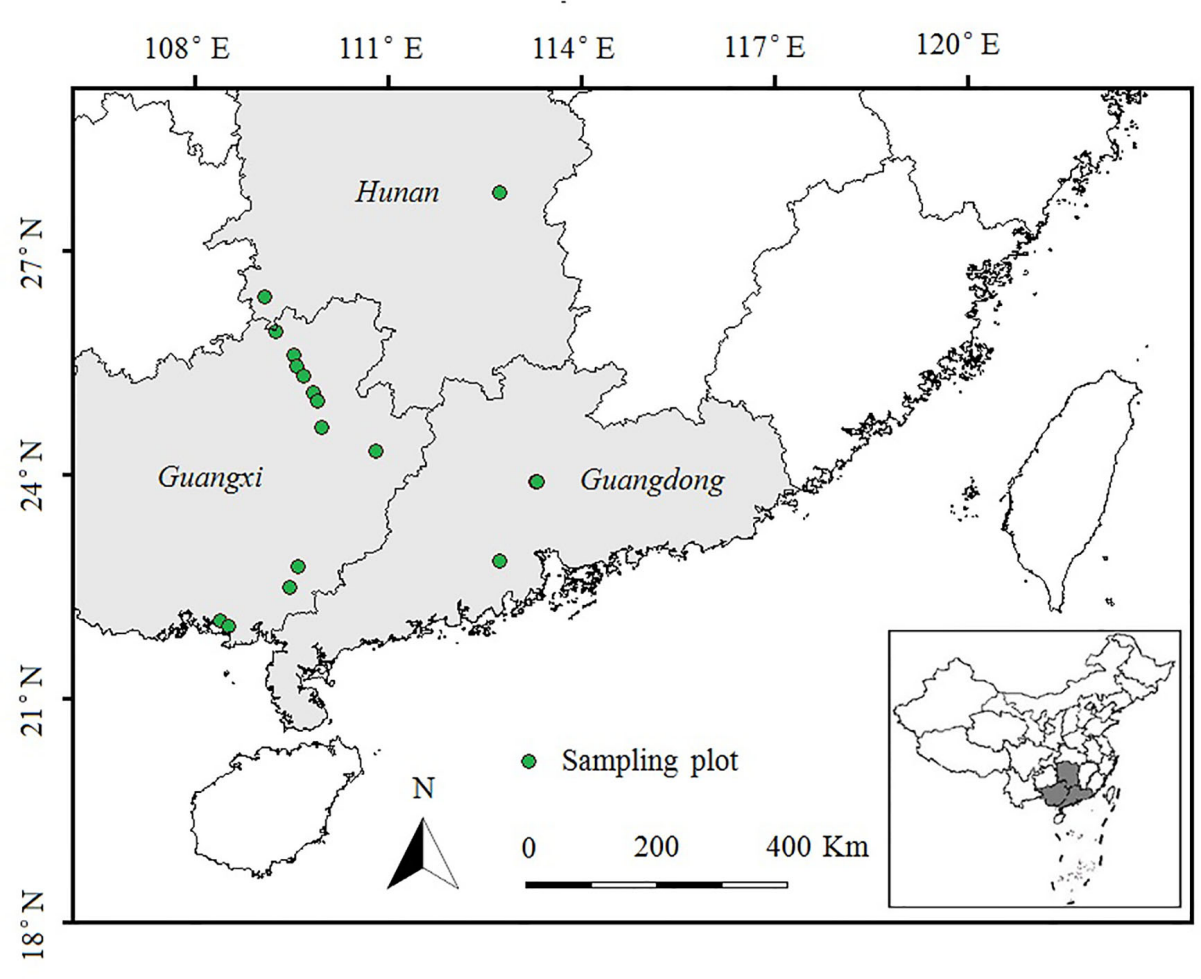
Sampling plots in *E. crassipes*-invaded areas in southern China along latitudinal gradients (21°N to 28°N). Plot size: 10 × 10 m; Transect size: 10 m; Quadrat size: 0.5 × 0.5 m.

During July and August 2014, we conducted a field survey of invasive *E. crassipes* in Hunan, Guangxi and Guangdong Provinces in southern China. We selected ponds or rivers in which *E. crassipes* covered an area of more than 100 m^2^. We set 20 plots (10 m × 10 m, at least 10 km apart) spanning a latitudinal gradient from 21°N to 28°N (as shown in **Figure 1**). Three 10 m transects were evenly set up in each plot, and five quadrats (0.5 m × 0.5 m) were uniformly spaced 2 m apart in each transect. We used the measurement methods of Wu et al. (2017) to record the species name, height, coverage, and abundance of each plant species in every quadrat. After the investigation of the plant community, we randomly collected the aboveground portions of eight *E. crassipes* individuals from each plot and stored them separately in sealed plastic bags filled with silicone. These plant samples were then brought back to the laboratory to measure the total carbon and total nitrogen contents of the leaves and stems of *E. crassipes* by using a vario TOC cube analyzer (Elementar, Germany), and the stoichiometric C:N ratios (total carbon/total nitrogen) in the leaves and stems were calculated.

We recorded the longitude, latitude and elevation within each plot using a handheld GPS receiver (Garmin eTrex 20, Kansas, USA). The conductivity and dissolved oxygen and nitrate nitrogen contents of the water were measured using a YSI water quality analyzer (Professional Plus, Ohio, USA). We successively calibrated the nitrate nitrogen sensor of the YSI analyzer with 100 mg/L and 1 mg/L standard solutions before measuring the water quality. The data probes were inserted below the water surface at a depth of 20 cm, and the data were read when a stable display was observed (Wu et al., 2017; Budzyńska et al., 2019). We obtained and calculated accurate data for the mean annual temperature and mean annual precipitation of every sampling plot in the last 50 years from the database of the National Meteorological Center, China Meteorological Administration (http://www.nmc.cn/) (the values of environmental factors in each plot are shown in **Supplementary Table 1**).

### Data Analysis

Based on the field survey, the relative importance value (*IV*) of the plant species in each plot was calculated using the following formula:

Relative IV=(relative cover+relative height+relative abundance)/3

while the total *IV* was the sum of a plant species’ relative *IV* in all plots (Jing et al., 2014).

To assess the plant diversity of the *E. crassipes-*invaded community, the following four *α*-species diversity indices were calculated (Higuti et al., 2007; Wu et al., 2016):

Patrick richness index: R=S;

$$\text{Simpson diversity index: }\lambda =1-\Sigma {\text{P}}_{i}^{2};$$

$$\text{Shannon-Wiener diversity index: }H=\text{-}\Sigma {\text{P}}_{i}\text{ ln }{\text{P}}_{i};$$

Pielou evenness index: E=(-ΣP_i_ ln P_i_)/ln S, where S is the total number of plant species in a plot, and P_i_ is the relative abundance of species i.

We used regression analysis to examine the relationships between eight environmental factors (longitude, latitude, elevation, conductivity, dissolved oxygen, nitrate nitrogen, temperature, and precipitation) and the nine biotic traits of *E. crassipes* (abundance, height, species cover, the total carbon content of the leaves, and stems, the total nitrogen content of the leaves and stems, and the C:N ratio of the leaves and stems). We applied 11 regression models (linear, quadratic, compound, growth, logarithmic, cubic, *S*, exponential, inverse, power, and logistic) provided in SPSS 16.0 software (SPSS Inc., Chicago, USA). Then, we selected the best-fitting models showing the maximum fitting coefficient (*R^2^*) and a significant *P* value (*P* < 0.05). We used the same method to examine the relationship between the relative *IV* of *E. crassipes* and four species diversity indices.

To examine the effect of environmental heterogeneity on the plant diversity and species distribution within communities invaded by *E. crassipes*, we first established an environmental matrix containing eight environmental factors (20 × 8), with a plant diversity matrix containing four indices (20 × 4) and a relative *IV* matrix containing 15 plant species (20 × 15). We then conducted detrended correspondence analysis (DCA) using the quantitative ordination software Canoco 4.5 (Microcomputer Power, New York, USA) to predict whether the linear or unimodal model would be appropriate for use. The results indicated that both of the longest gradient lengths of the plant diversity matrix and relative *IV* matrix were less than three standard deviations, thus, we preferred to perform redundancy analysis (RDA) (Chappuis et al., 2014). We also established an *E. crassipes* invasion matrix containing nine biotic traits (20×9) and used the same method to examine the effect of *E. crassipes* invasion on plant diversity. The variable in the RDA ordination diagram that presented the highest value of the correlation coefficient was indicated to be the main determinant factor of this ordination axis. We performed a Monte Carlo permutation test based on 499 random permutations to test the significance of the correlation coefficient (Ter Braak and Smilauer, 2002).

## Results

### Environmental Determinants of *E*. *crassipes* Performance

**Figure 2:**
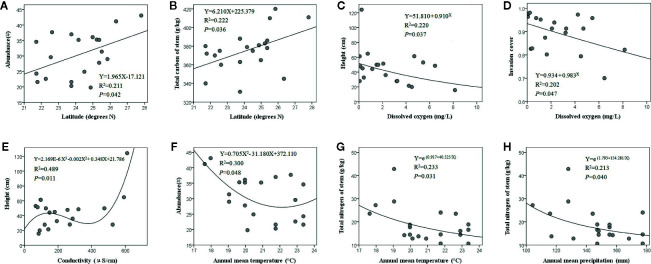
The abiotic environmental factors which significantly affected *E. crassipes* performance are latitude **(A, B)**, dissolved oxygen **(C, D)**, conductivity **(E)**, annual mean temperature **(F, G)** and annual mean precipitation **(H)**.

The optimal fitting relationships between latitude and *E. crassipes* abundance (*F*_1, 18_ = 4.817, *P* = 0.042) and the total carbon content of the stem (*F*_1, 18_ = 5.124, *P* = 0.036) were all linear equations, and with increasing latitude, these two traits showed significant increases (**Figures 2A, B**). Dissolved oxygen presented significant compound regression relationships with the height (*F*_1, 18_ = 5.078, *P* = 0.037) and invasion cover (*F*_1, 18_ = 4.545, *P* = 0.047) of *E. crassipes*, with both parameters decreasing with increasing dissolved oxygen levels (**Figures 2C, D**). The variation in *E. crassipes* height along the conductivity gradient was unstable and was characterized by a cubic equation (*F*_2, 17_ = 5.114, *P* = 0.011); however, *E. crassipes* height overall increased with increasing conductivity (**Figure 2E**). The optimal fitting relationships between the annual mean temperature and *E. crassipes* abundance (*F*_2, 17_ = 3.637, *P* = 0.048) and the total nitrogen content of the stem (*F*_1, 18_ = 5.455, *P* = 0.031) were represented by quadratic and *S* equations, respectively. Increasing temperature significantly decreased abundance and the stem nitrogen content (**Figures 2F, G**). Increased annual mean precipitation also significantly reduced the stem nitrogen content of *E. crassipes*, which was shown by the *S* equation (*F*_1, 18_ = 4.874, *P* = 0.040) (**Figure 2H**). In addition, elevation and the nitrate nitrogen content of water had no significant effect on *E. crassipes* performance, and all eight environmental factors had no significant effects on the C:N value of *E. crassipes.*

### Effect of Environmental Factors on Plant Diversity

A total of 15 plant species in eight families and 14 genera were recorded in our 20 plots. Poaceae exhibited the greatest richness, with five species in our plots, followed by Pontederiaceae and Polygonaceae, which both presented two species (see **Supplementary Table 2**). Among all species in the communities, *E. crassipes* exhibited the greatest total importance value (*IV* = 17.818). The other primary accompanying species based on the total *IV* were *Paspalum distichum* (*IV* = 0.660), *Monochoria vaginalis* (*IV* = 0.347) and *A. philoxeroides* (*IV* = 0.279). The ranges of the four species diversity indices in each plot were as follows: Patrick richness index (1–5), Simpson diversity index (0–0.554), Shannon–Wiener diversity index (0–1.056), and Pielou evenness index (0-0.675).

**Figure 3:**
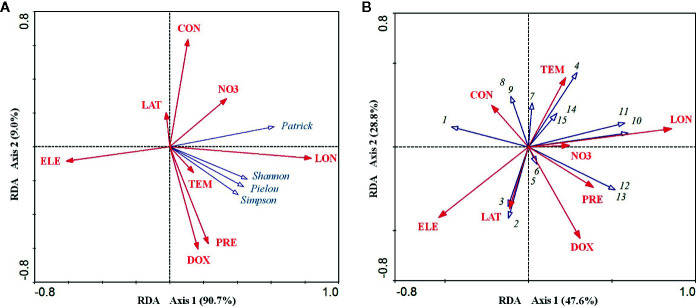
RDA ordination diagrams of eight abiotic environmental factors and four species diversity indices **(A)** and 15 plant species in the *E. crassipes* community **(B)**. LAT, LON, ELE, NO3, DOX, CON, TEM, and PRE represent latitude, longitude, elevation, nitrate nitrogen, dissolved oxygen, conductivity, mean annual temperature, and mean annual precipitation, respectively. Arabic numerals represent the 15 plant species as follows: 1 *Eichhornia crassipes*, 2 *Paspalum distichum*, 3 *Monochoria vaginalis*, 4 *Alternanthera philoxeroides*, 5 *Beckmannia syzigachne*, 6 *Arthraxon hispidus*, 7 *Polygonum sieboldii*, 8 *Echinochloa phyllopogon*, 9 *Hydrocharis dubia*, 10 *Oryza sativa*, 11 *Acorus calamus*, 12 *Fimbristylis dichotoma*, 13 *Leersia hexandra*, 14 *Polygonum hydropiper*, and 15 *Humulus scandens*.

For the relationship between the environmental factors and species diversity indices, the RDA ordination results showed that the cumulative percentage of variance in the diversity–environment relationships explained by the first two canonical axes reached 99.70% (90.70% for axis1 and 9.00% for axis2). Among these eight environmental factors, longitude was significantly positively related to axis1 (coefficient = 0.837, *P* < 0.001) and elevation was significantly negatively related to axis1 (coefficient = −0.612, *P* < 0.01). Dissolved oxygen (coefficient = −0.606, *P* < 0.01) and precipitation (coefficient = −0.573, *P* < 0.01) were both significantly negatively related to axis2, while conductivity was significantly positively related to the axis2 (coefficient = 0.635, *P* < 0.01) (**Table 1**). However, longitude and elevation were the most important drivers of plant diversity in the *E. crassipes*-invaded community, as the loading on axis1 was much higher than that on axis2. Longitude was highly positively correlated with the four species diversity indices, particularly the Patrick richness index, while elevation was strongly negatively correlated with all of the diversity indices (**Figure 3A**). Dissolved oxygen and precipitation were both positively correlated with the Simpson diversity index, Pielou evenness index and Shannon–Wiener diversity index but presented very week correlations with the Patrick richness index (**Figure 3A**).

**Table 1:** Correlations between the eight abiotic environmental factors and the first two RDA axes.

Environment factors	Environment–diversity indices		Environment–species distribution	
	Axis 1	Axis 2	Axis 1	Axis 2
LAT	−0.022	0.202	−0.114	−0.369
LON	0.837***	−0.068	0.858***	0.109
ELE	−0.612**	−0.085	−0.531*	−0.422
NO3	0.336	0.284	0.246	0.008
DOX	0.167	−0.606**	0.310	−0.547*
CON	0.108	0.635**	−0.221	0.248
TEM	0.141	−0.152	0.222	0.414
PRE	0.227	−0.573**	0.389	−0.242

LAT, LON, ELE, NO3, DOX, CON, TEM, and PRE represent latitude, longitude, elevation, nitrate nitrogen, dissolved oxygen, conductivity, mean annual temperature, and mean annual precipitation, respectively.

*P < 0.05 level. **P < 0.01 level. ***P < 0.001 level.

### Effect of Environmental Factors on Species Distributions

For the relationship between the environmental factors and species distributions, the cumulative percentage variance explained by the first two canonical RDA axes was 76.40% (47.60% for axis1 and 28.8% for axis2, see **Figure 3B**). Longitude was significantly positively related to axis1 (coefficient = 0.858, *P*<0.001) and elevation was significantly negatively related to axis1 (coefficient = −0.531, *P* < 0.05), while dissolved oxygen was significantly negatively related to axis2 (coefficient = −0.547, *P* < 0.05) (**Table 1**). In the RDA biplot (**Figure 3B**), *E. crassipes* (1) was strongly negatively correlated with longitude and dissolved oxygen but slightly positively correlated with elevation. *Oryza sativa* (10) and *Acorus calamus* (11) were both strongly positively correlated with longitude. *P. distichum* (2) and *M. vaginalis* (3) were positively correlated with elevation, while *A. philoxeroides* (4), *Polygonum hydropiper* (14) and *Humulus scandens* (15) were all strongly negatively correlated with elevation. The species exhibiting strong correlations with high dissolved oxygen were *Beckmannia syzigachne* (5) and *Arthraxon hispidus* (6), while *Echinochloa phyllopogon* (8) and *Hydrocharis dubia* (9) were highly negatively correlated with dissolved oxygen. In addition, *Fimbristylis dichotoma* (12), *Leersia hexandra* (13), *O. sativa* (10), and *A. calamus* (11) were extremely negatively correlated with *E. crassipes*, as their vector directions in our RDA biplot were almost opposite (**Figure 3B**).

### Relationship Between *E*. *crassipes* Invasion and Plant Diversity

**Figure 4:**
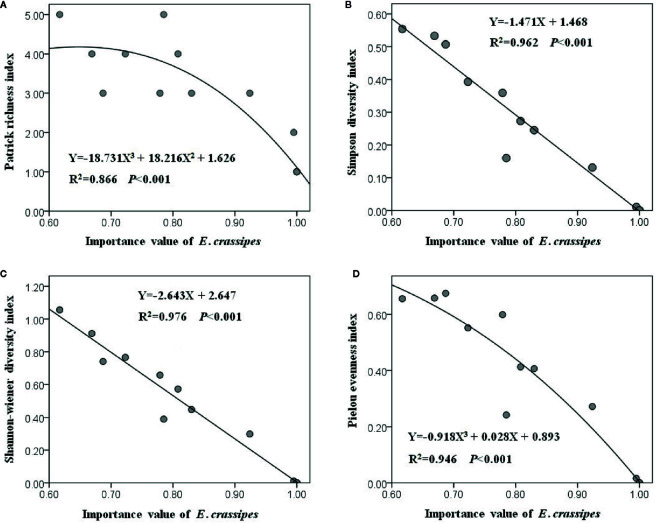
The comprehensive diversity **(B, C)** showed the faster downward tendency than species richness **(A)** and community evenness **(D)** with the increasing *E. crassipes* invasion.

All of the 20 plots were dominated by *E. crassipes*, and the relative *IV* of *E. crassipes* in each plot varied from 0.617 to 1.000 with an average value of 0.891. The optimal fitting relationships between the relative *IV* of *E. crassipes* and the Patrick richness index (*F*_2, 17_ = 54.880, *P* < 0.001) and Pielou evenness index (*F*_2, 17_ = 147.595, *P* < 0.001) were all represented by cubic equations, while the optimal fitting relationships between the relative *IV* of *E. crassipes* and the Simpson (*F*_1, 18_ = 457.780, *P* < 0.001) and Shannon–Wiener diversity indices (*F*_1, 18_ = 718.332, *P* < 0.001) were all linear equations (**Figure 4**). With an increase in the *E. crassipes* *IV*, all four diversity indices decreased; however, the Simpson and Shannon–Wiener diversity indices showed a faster downward tendency (**Figures 4B, C**), indicating that *E. crassipes* invasion caused greater negative effects on the comprehensive diversity than species richness and community evenness.

**Figure 5:**
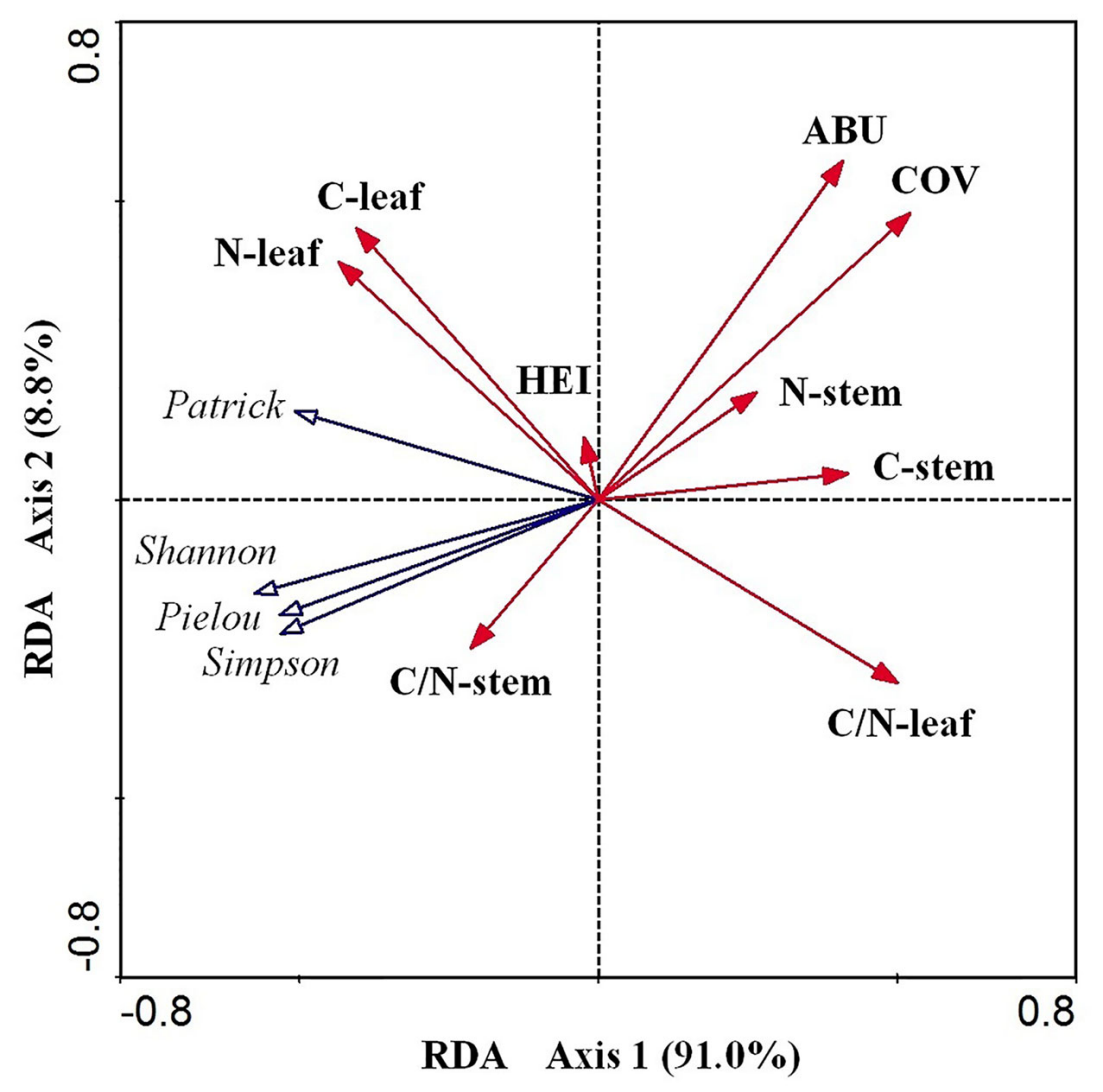
RDA ordination diagram of the nine biotic traits of *E. crassipes* and four species diversity indices. C-leaf, N-leaf, C-stem, N-stem, ABU, HEI, COV, C/N-leaf, and C/N-stem represent the total carbon content of the leaves, the total nitrogen content of the leaves, the total carbon content of the stem, the total nitrogen content of the stem, abundance, height, *E. crassipes* coverage, C:N of the leaves, and C:N of the stem, respectively.

According to the RDA results (**Table 2** and **Figure 5**), the cumulative percentage of variance explaining the *E. crassipes* traits–diversity index relationships of the first two canonical axes reached 99.80% (91.0% for axis1 and 8.80% for axis2). Species cover and leaf C:N were the primary biotic determinants of plant diversity, which were all significantly positively related to axis1 (coefficient = 0.522, *P* < 0.05; coefficient = 0.501, *P* < 0.05, respectively). The abundance of *E. crassipes* was significantly positively related to axis2 (coefficient = 0.567, *P* < 0.05). The Simpson and Shannon–Wiener diversity and Pielou evenness index and were strongly negatively correlated with *E. crassipes* cover and abundance, while the Patrick richness index was slightly negatively correlated with these two traits. The four diversity indices were also strongly negatively correlated with the leaf C:N ratio of *E. crassipes*, especially the Patrick richness index (**Figure 5**). The remaining other traits exhibited no significant relationship with the RDA axes.

**Table 2:** Correlations between the nine biotic traits of *E. crassipes* and the first two RDA axes.

Environment factors	Traits of *E. crassipes*-diversity indices	
	Axis 1	Axis 2
C-leaf	−0.405	0.454
N-leaf	−0.435	0.397
C-stem	0.418	0.044
N-stem	0.264	0.180
ABU	0.409	0.567*
HEI	−0.024	0.104
COV	0.522*	0.480
C/N-leaf	0.501*	−0.307
C/N-stem	−0.213	−0.248

C-leaf, N-leaf, C-stem, N-stem, ABU, HEI, COV, C/N-leaf, and C/N-stem represent the total carbon content of the leaves, the total nitrogen content of the leaves, the total carbon content of the stem, the total nitrogen content of the stem, abundance, height, invasion cover, the C:N ratio of the leaves, and the C:N ratio of the stem, respectively.

*P < 0.05 level.

## Discussion

In this study, we identified the abiotic and biotic determinants of plant diversity associated with *E. crassipes* invasion in southern China. We found that all four calculated α-species diversity indices significantly decreased with the increase in *E. crassipes* importance value. Increasing latitude increased *E. crassipes* abundance and the total carbon content of the stem. Increasing *E. crassipes* height and species cover could reduce the dissolved oxygen, while increase in water conductivity increased *E. crassipes* height. We also found that elevated temperature decreased *E. crassipes* height and the total nitrogen content of the stem, and elevated precipitation also decreased stem nitrogen. The RDA results showed that the primary environmental determinants of plant diversity in the *E. crassipes* community were longitude and elevation, while the biotic determinants associated with *E. crassipes* traits were *E. crassipes* cover, the leaf C:N ratio and abundance.

Invasive plants usually exhibit greater phenotypic plasticity and photosynthetic abilities than accompanying native plants (Godoy et al., 2012; Wang et al., 2017). In higher latitudes with relatively extreme environments, many invasive plants may show changes in their ecological and/or physiological traits in response to dramatic environmental changes (Lacoul and Freedman, 2006; Wu et al., 2017), as observed in *E. crassipes* in our study. In general, increasing latitude decreases solar irradiance, temperature and precipitation (Santamaría, 2002). Thus, as a thermophilous invasive plant native to the tropics, a higher *E. crassipes* population density confers the advantage of capturing more solar energy in higher latitudes comparing with the native cooccurring plants, which could compensate for the energy loss caused by thermal fluctuations (Liu et al., 2016). An increase in stem carbon could improve individual toughness and tolerance to stresses by increasing the biosynthesis of lignins and polyphenols, because lignins depositing in secondary cell wall of stem tissues can improve the structural support and water transport in plant growth and development. Thus, in colder environments increasing lignins could increase stem hardness and then enhance plant tolerance (Cabané et al., 2012; Liu et al., 2018). Polyphenols as important secondary chemicals responding to abiotic stresses may also be associated with invasive plant tolerance to low temperature (Sharma et al., 2019). Thus, increasing carbon might facilitate the establishment and spread of *E. crassipes* in stressed habitats in higher latitudes. Additionally, the obvious seasonal exchange of atmospheric CO_2_ in higher latitudes would also stimulate the carbon concentration of plant tissues (Denning et al., 1995; Marko et al., 2008; Elser et al., 2010). The similar patterns of abundance and stem carbon varied along the latitudinal gradient have been observed in other aquatic invasive plants, such as *A. philoxeroides* in China and *P. australis* in Canada (Cronin et al., 2015; Wu et al., 2017). As there was a significant negative co-varying relationship between latitude and temperature at large spatial scales, the response of *E. crassipes* abundance to increasing temperature showed an opposite trend to that of the latitude gradient (**Figure 2F**).

According to the latitudinal biotic interaction hypothesis (Schemske et al., 2009; Moles et al., 2011), plants may suffer more pressures from herbivores at lower latitudes. Thus, plants at low latitudes may develop high resistance to herbivores. This could explain the lower nitrogen contents in plant tissue in low latitudes (**Figures 2G, H**), for reducing its palatability for herbivores (Schemske et al., 2009; Grutters et al., 2017). Some relevant studies also have shown that relative to the plants that grow under decreased annual mean temperature and precipitation in high latitudes, aquatic invasive plants in low latitudes are less nutritious (Morrison and Hay, 2012; Cronin et al., 2015; Petruzzella et al., 2017). The vigorous growth of aquatic invasive plants and the decomposition of their litter usually consume a large amount of dissolved oxygen, which greatly restricts native plant growth, while higher dissolved oxygen levels in water environments might benefit the restoration of the native plant community by alleviating competition for oxygen and, thus, decrease invasive plant competitiveness (Sakurai et al., 2017; Wu and Ding, 2019), explaining the decreasing height and plant cover of *E. crassipes* with increasing dissolved oxygen levels. Conductivity is usually proportional to inorganic ion concentrations (e.g. Na^+^, Mg^2+^, Cl^−^); therefore, as a result of the salt-induced water deficit, plant height usually decreases with increased conductivity (Wang et al., 2019). However, many aquatic invasive plants exhibit high salt tolerance (such as *Myriophyllum spicatum*, *P. australis* and *E. crassipes*). Thus, increased water conductivity may stimulate vigorous compensatory growth, resulting in a greater plant height (Thouvenot et al., 2012; Chappuis et al., 2014; Stoler et al., 2018). Nitrate nitrogen had no effect on *E. crassipes* performance in this study, likely because *E. crassipes* has a high nitrogen adsorption capacity (Gao et al., 2013; You et al., 2014), and our plots showed relatively high contents of nitrate nitrogen.

A previous study showed species diversity of hydrophytes increases from the northwest to the southeast with increasing longitude in China (Li et al., 2006), and this pattern was applied to the *E. crassipes*-invaded communities in this study. It might be related to changes in water salinity and hydrological variability along the longitudinal gradient. Moreover, the diverse rugged aquatic landscapes and water-level fluctuations caused by the variable rainfall patterns across longitudes may also support higher hydrophyte diversity and thus resist aquatic plant invasions (Goldblatt, 1997; Alahuhta et al., 2018). With an increase in elevation, habitat filtering plays a stronger role in shaping the biological community assemblage, especially for the aquatic plant community (Laliberte et al., 2014; Liu and Wang, 2018). A phenomenon similar to the higher species diversity of hygrophytes in lower-elevation regions observed in our *E. crassipes* community has been found in the Yangtze River of China (Liu and Wang, 2018). Elevated dissolved oxygen and precipitation could alleviate the interspecific competition between invasive and native aquatic plants (Ji et al., 2009; Lacet et al., 2019), thus increasing the Shannon–Wiener and Simpson diversity indices and Pielou evenness index. However, as a measure of salinity, conductivity has been proven to have strong detrimental effects on native hydrophyte diversity but to facilitate aquatic alien plant invasions, as these invasive plants are always salt tolerant (Nielsen et al., 2003; Pulzatto et al., 2019). The Patrick richness index was weakly affected by the water environment, likely due to the low plant richness in the invaded community. In fact, in some sampling plots, *E. crassipes* was the only species present; thus, the Patrick richness index was insensitive to microhabitat changes. In the RDA biplot, *Oryza sativa*, *A. calamus*, *F. dichotoma*, and *L. hexandra* showed exerted negative effects on *E. crassipes*, likely due to their superior properties related to the resistance of biological invasions, such as taller growth or tillering growth, allowing them to coexist for a long time in this invaded community. Consistent with our previous studies on aquatic *A. philoxeroides-*invaded community (Wu et al., 2017), in this study, we found the plant diversity of the *E. crassipes* community increased as longitude increased. These findings have important implications for predicting aquatic plant invasions and their effects on plant diversity. For example, the rainfall in mainland China would continuously increase with increasing longitude under ongoing climate change, which my further improve aquatic invasive plant growth (Liu et al., 2008; Anufriieva and Shadrin, 2018; Wu and Ding, 2019). Additionally, more frequent anthropogenic activities in high longitude in China could result in more pollutants, *e.g.*, organic compounds, nitrogen, and phosphorus, subsequently leading to water eutrophication and then facilitating aquatic invasive plant invasions (Ding et al., 2008; Le et al., 2010; Wu and Ding, 2019). Together, all the above-mentioned factors could trigger aquatic plant invasions and then negatively change plant diversity of aquatic invaded communities across longitudes. Therefore, more attention should be paid to the species diversity of aquatic invasive plant communities along a longitudinal gradient at large biogeographical scales compared to the LDG rule (latitudinal diversity gradient) of their terrestrial counterparts (Wu et al., 2016).

The vigorous asexual ramets (abundance) of aquatic invasive plants allow them to rapidly and fully occupy the available niche space, limiting the establishment of other native aquatic plants and, thus, dramatically decreasing aquatic plant diversity (Stiers and Triest, 2017; Yu et al., 2019). However, in our study, height did not contribute to *E. crassipes* invasiveness, likely because there were several tall hydrophytes in the invaded community (*e.g.*, *P. distichum*, *B. syzigachne*, *A. calamus*, *L. hexandra*), which could disrupt the shading effect of *E. crassipes.* The plant C:N ratio may be correlated with defense ability, affecting plant diversity in invaded communities (Sardans et al., 2017). An increasing C:N ratio usually leads to higher total phenolic contents and leaf toughness of invasive plants, which would decrease plant nutritional quality and, thus, reduce palatability to herbivores, facilitating invasiveness and increasing the negative impacts on native species (Cronin et al., 2015; Petruzzella et al., 2017; Zerche et al., 2019). This is consistent with the negative effect of a higher leaf C:N ratio of *E. crassipes* on native plant diversity observed in our study. Similar phenomena have been found in other aquatic invasive plants such as *Spartina alterniflora* in China and *P. australis* in Europe (Cronin et al., 2015; Qiao et al., 2018). The C:N ratio of *E. crassipes* was weakly affected by the abiotic environment, and this internal stability of ecological stoichiometry might be one of the important mechanisms contributing to aquatic plant invasions (Gonzalez et al., 2010).

In summary, in this study, we identified the abiotic and biotic determinants of plant diversity in *E. crassipes*-invaded communities. These findings also provide insights for the risk assessment of other aquatic invasive plants. The spread and establishment of many aquatic invasive plants may further accelerate under rapid global change (Wu and Ding, 2019), and aquatic ecosystems would thus suffer a more serious invasion threat (Capers et al., 2007; Wu et al., 2017). In the context of global warming and the northward shift of rain belts, the prediction and assessment of aquatic invasive plants will become more complicated at higher latitudes. Exploring the determinants and ecological effects of these aquatic invasive plants is crucial for predicting their dynamics in a changing environment and prioritizing biodiversity protection efforts.

## Data Availability Statement

All datasets presented in this study are included in the article/**Supplementary Material**.

## Author Contributions

JD and HW designed the study. HW conducted the experiment and analyzed the data. HW wrote the first draft of the manuscript. HW and JD performed a critical revision and approved the final version to be published.

## Funding

This study was funded by the National Key Research and Development Program (2017YFC1200100), the National Natural Science Foundation of China (31800460), and the Nanhu Scholars Program for Young Scholars of Xinyang Normal University (XYNU).

## Conflict of Interest

The authors declare that the research was conducted in the absence of any commercial or financial relationships that could be construed as a potential conflict of interest.
