# Supplementary material for: Abiotic and Biotic Determinants of Plant Diversity in Aquatic Communities Invaded by Water Hyacinth [Eichhornia crassipes (Mart.) Solms]
Source: Front Plant Sci. 2020 Aug 25;11:1306. doi: 10.3389/fpls.2020.01306 (PMC7477091; doi:10.3389/fpls.2020.01306)
Supplement: Supplementary file 1 [file Table_1.docx]

**Supplementary Table 1.** The values of environmental factors in each plot invaded by *E. crassipes*

| Plot  No. | Latitude  / °N | Longitude  / °E | Elevation  / m | N-NO_3_^-^  / mg^.^kg^-1^ | D-Oxygen  / mg^.^L^-1^ | Conductivity  / μS^.^cm^-1^ | Temperature  / °C | Precipitation  / cm |
| --- | --- | --- | --- | --- | --- | --- | --- | --- |
| 1 | 25.842 | 109.799 | 183 | 0.490 | 5.45 | 66.4 | 19.045 | 127.927 |
| 2 | 25.513 | 110.066 | 229 | 0.310 | 0.31 | 179.6 | 19.045 | 127.927 |
| 3 | 26.347 | 109.651 | 326 | 0.230 | 8.15 | 84.5 | 17.628 | 115.846 |
| 4 | 25.358 | 110.102 | 203 | 0.270 | 1.13 | 149.1 | 19.968 | 155.360 |
| 5 | 25.358 | 110.101 | 201 | 0.080 | 1.50 | 127.1 | 19.968 | 155.360 |
| 6 | 25.223 | 110.193 | 164 | 0.370 | 2.07 | 77.8 | 19.642 | 157.447 |
| 7 | 24.982 | 110.341 | 177 | 0.730 | 2.27 | 286.2 | 19.968 | 155.360 |
| 8 | 24.867 | 110.384 | 158 | 1.050 | 6.46 | 320.7 | 20.116 | 131.368 |
| 9 | 24.502 | 110.448 | 110 | 2.710 | 0.75 | 591.0 | 20.466 | 116.743 |
| 10 | 24.163 | 111.210 | 121 | 1.180 | 3.26 | 269.3 | 20.911 | 168.254 |
| 11 | 21.773 | 109.030 | 40 | 0.860 | 1.64 | 474.8 | 23.365 | 147.161 |
| 12 | 21.712 | 109.135 | 3 | 0.580 | 0.23 | 610.0 | 23.365 | 147.161 |
| 13 | 21.704 | 109.145 | 4 | 0.390 | 4.24 | 141.8 | 23.365 | 147.161 |
| 14 | 22.257 | 109.997 | 64 | 0.120 | 0.42 | 192.3 | 22.901 | 148.117 |
| 15 | 22.548 | 110.115 | 84 | 0.190 | 0.07 | 97.4 | 22.901 | 148.117 |
| 16 | 22.624 | 112.954 | 35 | 0.800 | 3.21 | 120.1 | 22.635 | 144.150 |
| 17 | 23.744 | 113.462 | 30 | 0.280 | 4.49 | 90.5 | 21.755 | 177.417 |
| 18 | 23.747 | 113.486 | 39 | 0.160 | 0.12 | 255.7 | 21.755 | 177.417 |
| 19 | 23.747 | 113.486 | 43 | 1.350 | 4.70 | 95.5 | 21.755 | 177.417 |
| 20 | 27.807 | 112.954 | 46 | 2.600 | 0.07 | 524.0 | 17.974 | 104.251 |
