# Supplementary material for: Abiotic and Biotic Determinants of Plant Diversity in Aquatic Communities Invaded by Water Hyacinth [Eichhornia crassipes (Mart.) Solms]
Source: Front Plant Sci. 2020 Aug 25;11:1306. doi: 10.3389/fpls.2020.01306 (PMC7477091; doi:10.3389/fpls.2020.01306)
Supplement: Supplementary file 2 [file Table_2.docx]

**Supplementary Table 2.** The total importance values of 15 plant species in *E. crassipes* community

| Code | Species name | Family | Total *IV* |
| --- | --- | --- | --- |
| 1 | *Eichhornia crassipes* | Pontederiaceae | 17.818 |
| 2 | *Paspalum distichum* | Poaceae | 0.660 |
| 3 | *Monochoria vaginalis* | Pontederiaceae | 0.347 |
| 4 | *Alternanthera philoxeroides* | Amaranthaceae | 0.279 |
| 5 | *Beckmannia syzigachne* | Poaceae | 0.178 |
| 6 | *Arthraxon hispidus* | Poaceae | 0.175 |
| 7 | *Polygonum sieboldii* | Polygonaceae | 0.109 |
| 8 | *Echinochloa phyllopogon* | Poaceae | 0.102 |
| 9 | *Hydrocharis dubia* | Hydrocharitaceae | 0.098 |
| 10 | *Oryza sativa* | Poaceae | 0.063 |
| 11 | *Acorus calamus* | Araceae | 0.056 |
| 12 | *Fimbristylis dichotoma* | Cyperaceae | 0.036 |
| 13 | *Leersia hexandra* | Poaceae | 0.032 |
| 14 | *Polygonum hydropiper* | Polygonaceae | 0.028 |
| 15 | *Humulus scandens* | Moraceae | 0.021 |
